# Socio-demographic characteristics are related to the advanced clinical stage of oral cancer

**DOI:** 10.4317/medoral.23105

**Published:** 2019-10-27

**Authors:** Larissa Suelen da Silva Lins, Natanael Victor Furtunato Bezerra, Aldelany Ramalho Freire, Leopoldina de Fátima Dantas de Almeida, Edson Hilan Gomes de Lucena, Yuri Wanderley Cavalcanti

**Affiliations:** 1 School of Dentistry. Federal University of Paraíba (UFPB), João Pessoa, Brazil; 2 Department of Clinical and Social Dentistry. Graduate Program in Dentistry. Federal University of Paraíba (UFPB), João Pessoa, Brazil

## Abstract

**Background:**

Social determinants may be associated with the onset and progression of the clinical stage of oral cancer. Aim: To evaluate the impact of socio-demographic characteristics on the prevalence of advanced clinical stage of oral cancer.

**Material and Methods:**

Information about 51,116 cases of oral cancer, from all Brazilian states, between 2000 and 2012, was obtained from the Cancer Registry Information System. The clinical stage of oral cancer (dependent variable) was classified as initial (stages I and II) or advanced (stages III and IV). The relationship between the clinical stage of oral cancer and the following independent variables was analyzed: sex, age, schooling, marital status, family history of cancer, and origin of referral. Analyses on frequency distribution and multivariate binary logistic regression model were performed (α<0.05).

**Results:**

Compared to individuals with no schooling, those who attended elementary to high school (OR=2.461) and college education (OR = 3.050) had a higher prevalence of advanced cases of oral cancer. Individuals without a partner (OR = 14,209) demonstrated a higher prevalence compared to married individuals. Subjects aged 20-44 years (OR = 4.081) and 45-64 years (OR = 14.875) had a higher prevalence compared to those aged 15-19 years. The variables gender, family history of cancer and origin of referral integrated the binary model of logistic regression, but did not present statistical significance.

**Conclusions:**

Socioeconomic factors may be related to the advanced clinical stage of oral cancer.

** Key words:**Mouth Neoplasms, Neoplasm Staging, Social Determinants of Health.

## Introduction

Oral malignancies are frequently found worldwide and within the Brazilian population, being the 12th most frequent tumor in Brazil (1). These are classified according to the TNM staging system recommended by the International Union Against Cancer (UICC) in four subtypes: I, II, III and IV. Classifications I and II corresponds to the initial stage with absence of metastasis; whilst stages III and IV correspond to advanced stages, in which metastatic components and detected (2,3).

The histological staging, the degree of tumor invasion and the occurrence of cervical metastases correspond to the factors that determine the prognosis of the disease and the postoperative management (1,2). The histological grade of the tumor evidenced by analyzes of lesion specimens, through a biopsy, can provide important information regarding the biological activity of the tumor cells, which directs the treatment to be instituted (4).

In addition to the biological factors of oral cancer, such as cellular aggressiveness, tumor size and presence of cervical metastases, socio-demographic health characteristics may also influence the incidence and severity of oral neoplasms (5-7). Oral squamous cell carcinoma is the most prevalent cancer type affecting oral tissues and advanced stage diagnosis of oral cancer negatively affects patient survival (8). Based on that, determining factors associated with advanced stage lesions are imperative to improve life expectancy of affected individuals.

Studies have suggested that there is a higher prevalence of head and neck cancer in socioeconomically vulnerable individuals, compared to those more privileged and with full access to medical services (9,10). Harmful habits such as smoking and drinking are considered risk factors for the development of oral cancer (2). In addition, these habits are more prevalent among the low-income population (11). Lack of dental insurance coverage also affects individuals' access to routine exams, resulting in delayed diagnosis, as most signs of oral cancers can be found through preventive dental clinical examination (12).

Individuals with low income and no health insurance are diagnosed when the disease is in a more advanced stage of aggression, usually tumors in larger size and associated with cervical metastases. Otherwise, those who have health insurance usually present the disease at an early stage, and consequently these have a better prognosis of survival (13).

Given the need for studies to verify the association of socioeconomic factors with the prevalence and progression of the clinical stage of oral cancer, the present study aimed to evaluate the distribution of cases of oral cancer and its relationship with social and economic factors in Brazil, from 2000 to 2012.

## Material and Methods

A cross-sectional study was carried out using secondary data from the Hospital Registry of Cancer Information System, linked to the National Cancer Institute of Brazil (https://irhc.inca.gov.br). This information system assembles data from all cases of cancer under diagnosis and treatment in Brazil. This dataset was extracted from a public information system, which does not require Ethical Committee approval. Patients gave informed consent at the moment of the consultation. Data collection was carried out between August and October 2017. Data from all cases of oral cancer was retrieved from the period between 2000 and 2012, and the following information was used: clinical stage of cancer, age group, schooling, marital status, family history of cancer, and origin of referral.

The information collected was based on the International Classification of Diseases for Oncology (ICD-10), in which the following cancer sites were selected: lips (C00), tongue base (C01), tongue (C02), gum (C03), mouth (C04), palate (C05), other non-specific parts of the mouth (C06), parotid gland (C07), other major salivary glands (C08), tonsils (C09) and oropharynx (C10). The histological type of cancer was not considered for analysis; however the prevalence of oral squamous cell carcinoma was set at 85%.

A total number of 74,842 cases of oral cancer were obtained, from which the inconsistent with no information data were removed. Final sample consisted of 51,116 cases of oral cancer, which were statistically analyzed. A multivariate binary logistic regression model was created, in which the clinical stage of oral cancer corresponded to the dependent variable. The clinical stage of cancer was categorized according to the TNM classification in: initial stage (I and II) - score 0, and advanced stage (III and IV) - score 1. The independent variables corresponded to the following variables: age group, schooling, marital status, family history of cancer, and origin of referral.

Data were tabulated and statistically analyzed using the software Statistical Package for Social Sciences (SPSS, v. 20, IBM, Chicago, IL), in which a multivariate binary logistic regression model was constructed (α<0.05). Variables with *p-value*<0,05 were considered statistically significant. The odds ratio and the 95% confidence interval were considered for the interpretation of results. Multivariate logistic regression calculated the chance of independent variables interfere with the prevalence of advanced stage of oral cancer.

## Results

There was a higher prevalence of advanced stages of oral cancer in males (78.4%), within the age group 45-64 years (57.2%), with elementary schooling (59.1%), and under single marital status (46%). According to the origin of the referral to the cancer diagnosis service, the highest prevalence (59.4%) was from the Brazilian Public Health System (SUS).

The highest prevalence of advanced stage of oral cancer was associated with age groups 20-44 years (OR = 4.081, *p-value* <0.001) and 45-64 years (OR = 14.875, *p-value* <0.001). Individuals over 65 years had a lower prevalence of advanced cases of cancer (OR = 0.019, *p-value* <0.001) compared to adolescents (15-19 years).

The schooling significantly interfered with the prevalence of advanced stage oral cancer; being observed that individuals with no schooling presented less frequency of advanced cases of cancer. In addition, the unmarried individuals (single, widowed and separated) presented higher chance to present advanced stage of oral cancer (OR = 14.209, *p-value* <0.001).

The variables family history of cancer and origin of referral integrated the multivariate binary logistic regression model, but did not present statistical significance with the prevalence of advanced cases of oral cancer (Table 1).

**Table 1:**
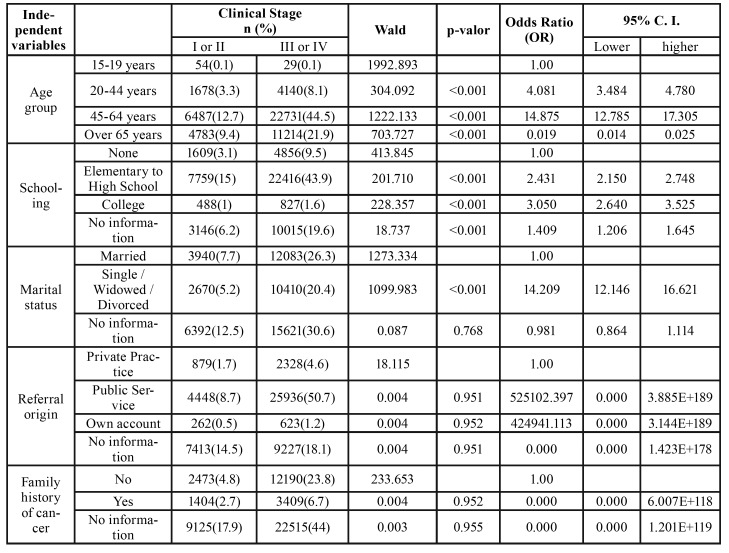
Distribution of the components of the sample according to the clinical stage (I / II or III / IV) and variables included within the adjusted multivariate logistic regression model (Age group, schooling, marital status, family history of cancer, and referral origin).

## Discussion

Prevalence of oral cancer is high and estimative in Brazil correspond to 11,200 new cases in men and the 3,500 new cases in women for each year (1). In addition, oral cancer is the tenth most frequent causes of death in the world (14,15). The present study demonstrates that socio-demographic characteristics are associated with a higher prevalence of advanced-stage lesions of oral cancer, some of them being schooling and age, already described in the literature in previous studies (3,16-18).

Our research team has previously demonstrated that alcohol and tobacco consumption significantly impact the prevalence of advanced stage oral cancer lesions (2). Our previous data show that tobacco is a significant risk factor for advanced stage lesions, being alcohol a modifying factor for smokers. This means that alcohol alone did not impact the prevalence of advanced stage oral cancer, whilst its association with tobacco increased significantly the prevalence of advanced stage lesions (2). The effect of deleterious habits was not considered in this analysis; but those should be considered together with the socioeconomic variables explored in the present study for the formulation of public health policies.

In the present study, we focused on the impact of socio-demographic characteristics. We choose not including deleterious habits in this study because it would significantly reduce the final sample size. As many of the registries do not include information on deleterious habits, roughly 30,000 cases would not be included in this analysis. The present study considered a final sample of 51,116 cases, in contrast with the previous study, which included 21,160 cases (2). Hereby, other important associated factors are explored in this study and they should account for public policies formulations directed to the prevention and early diagnosis of oral cancer (19).

Oral cancer is most often diagnosed at advanced stage, a fact contributes for lower patient survival and that can be justified by the lack of early diagnosis (8). As confirmed by the literature, most types of oral cancer consist of squamous cell carcinoma and the late diagnosis is frequently associated with the lack of knowledge of the signs, symptoms and causes of this disease (8). In the present study, squamous cell carcinoma consisted of 85% of all cases and diagnosis confirmation is frequently obtained in the advanced stage of the lesion, which contributes for lower patient survival (5-8).

The higher prevalence of advanced stage of oral cancer for individuals without partners (Unmarried, Single, Widowed, Divorced). can be explained by the fact that people engaged in marital relationships have more self-care and also a health support given by the partner, who tends to perceive the changes in the spouse and alerts him/her. The spouse would also act as an encouragement agent, which would guarantee greater chances of success in invasive treatments (20-23).

According to the age group, the group aged 45 to 64 years presented the higher prevalence for the development of advanced oral cancer. This fact can be explained due to cellular aging and the decrease of the regenerative capacity of the cells, together with greater exposure to carcinogenic factors, which would make them more susceptible to the development of tumors. However, it is important to point out that there is an increasing tendency for oral cancer to occur in the younger population (24).

According to the educational level, it was verified that the highest prevalence of oral cancer in advanced stages was in the group with higher education. This finding is in conflict with other literature results, which indicate that socioeconomically disadvantaged groups are related to higher rates of unemployment, low income and little access to education (25). However, the level of schooling should be considered alongside other factors related to the incidence of cancer, such as excessive consumption of alcohol, tobacco, sedentary lifestyle and irregular diet (26,27). In the present study, 25.8% of the sample presented inaccurate data on schooling ("no information"). In addition, 58.9% of the sample is concentrated in elementary to middle school. These aspects may have significantly influenced statistical probabilities and results should be analyzed with care.

This study presents data on socioeconomic factors that are associated with a higher prevalence of advanced cases of oral cancer. However, results should be analyzed with care, since analysis was based on secondary data, which may present failures related to their collection and feeding on the information system. Although several studies in the literature have used a cross-sectional approach to investigate cause and effect associations, it is recognized this is not the best study design for such purpose.

Regarding future perspectives, we suggest new assessments using other parameters to also estimate the effects of socioeconomic factors associated with the prevalence of the advanced stage of oral cancer. It is essential to consider that this study is representative of the Brazilian population, since, it includes data from the Information System of Cancer Hospital Records, over a 12-year period. Brazil has a continental and mixed population and the results of this study can serve as the basis for the population of other countries.

The present study demonstrates that socioeconomic factors may be related to the advancement of the clinical stage of oral cancer. Therefore, the importance of recognition of its clinical signs and early diagnosis should be better disseminated to the population to reduce the prevalence of unfavorable prognoses.
